# Fever-Range Hyperthermia Promotes Macrophage Polarization towards Regulatory Phenotype M2b

**DOI:** 10.3390/ijms242417574

**Published:** 2023-12-17

**Authors:** Henryk Mikołaj Kozłowski, Justyna Sobocińska, Tomasz Jędrzejewski, Bartosz Maciejewski, Artur Dzialuk, Sylwia Wrotek

**Affiliations:** 1 Department of Genetics, Faculty of Biological Sciences, Kazimierz Wielki University, 10 Powstańców Wielkopolskich Ave., 85-090 Bydgoszcz, Poland; dzialuk@ukw.edu.pl; 2 Department of Immunology, Faculty of Veterinary and Biological Sciences, Nicolaus Copernicus University, 1 Lwowska Str., 87-100 Torun, Poland; j.sobocinska@umk.pl (J.S.); tomaszj@umk.pl (T.J.);

**Keywords:** fever-range hyperthermia, macrophage polarization, fever, macrophages, inflammation

## Abstract

Fever-range hyperthermia (FRH) is utilized in chronic disease treatment and serves as a model for fever’s thermal component investigation. Macrophages, highly susceptible to heat, play a pivotal role in various functions determined by their polarization state. However, it is not well recognized whether this process can be modulated by FRH. To address this, we used two different macrophage cell lines that were treated with FRH. Next, to define macrophage phenotype, we examined their functional surface markers CD80 and CD163, intracellular markers such as inducible nitric oxide synthase (iNOS), arginase-1 (Arg-1), and the expression of interleukin-10 (IL-10) and tumor necrosis factor α (TNF-α). Additionally, in FRH-treated cells, we analyzed an expression of Toll-like receptor 4 (TLR-4) and its role in macrophage polarization. We also checked whether FRH can switch the polarization of macrophages in pro-inflammatory condition triggered by lipopolysaccharide (LPS). FRH induced M2-like polarization, evident in increased CD163, IL-10, and Arg-1 expression. Notably, elevated COX-2, TNF-α, and TLR-4 indicated potential pro-inflammatory properties, suggesting polarization towards the M2b phenotype. Additionally, FRH shifted lipopolysaccharide (LPS)-induced M1 polarization to an M2-like phenotype, reducing antimicrobial molecules (ROS and NO). In summary, FRH emerged as a modulator favoring M2-like macrophage polarization, even under pro-inflammatory conditions, showcasing its potential therapeutic relevance.

## 1. Introduction

Fever range hyperthermia (FRH) is a condition used in research to investigate a thermal component of fever [1,2]. FRH is also a medical procedure that increases the core body temperature to mimic fever. Despite reported benefits in the treatment of chronic diseases such as rheumatic diseases or cardiovascular disorders [3], the molecular changes induced by FRH remain poorly characterized. In our previous studies on mistletoe extract, we observed that macrophages are heat-sensitive cells that respond to heat with increased expression of pro-inflammatory cytokines such as interleukin (IL)-1β and IL-6 [4]. This observation is in accordance with other studies that demonstrated that elevated temperature can decrease the activation threshold required for the production of effector molecules in macrophages [5].

It is well established that macrophages play a decisive role in the detection, recognition, and neutralization of pathogens. They are involved in antigen presentation and initiation of immune response by releasing cytokines and chemokines that activate other immune cells [6]. Many antigens are sensed by macrophages through Toll-like receptors (TLRs), leading to the release of pro-inflammatory mediators such as cytokines or reactive oxygen species (ROS) and nitrogen oxide (NO) [7]. Among 10 known human TLRs, TLR-4 is of great biological significance due to its role in the initiation of immune response triggered not only by lipopolysaccharide (LPS) but also by damage-associated molecular patterns (DAMPs) [7,8]. Importantly, heat-shock proteins (HSPs) such as HSP-70, which, among others, are released in hyperthermic conditions, also have been identified as molecules that interact with TLR-4 [9]. Thus, it seems plausible that TLR-4 may be involved in heat-induced effects.

In response to various stimuli, macrophages can undergo polarization, which is a process that involves changes in their gene expression, morphology, and function [6]. In general, the heterogeneity of macrophages includes two main populations: classically activated M1 cells and alternatively activated M2 cells [10]. It is well-established that macrophages exhibit a wide array of receptors for a range of factors, encompassing growth factors, cytokines, and microbial products, with their activation and functional profiles within tissues being contingent upon the composite influence of these stimuli. In consequence, the conventional dichotomy of M1 and M2 activation models is being questioned since macrophages possess the capacity to swiftly adapt their phenotypes in response to the dynamic alterations in their microenvironment [11]. Classically activated M1 macrophages are commonly known as pro-inflammatory cells, which arise due to exposure to factors such as TLR-4 ligand (LPS) or in the presence of Th1 cytokines such as interferon gamma (IFN-γ) and granulocyte-macrophage colony-stimulating factor (GM-CSF) [10,12]. In a response to these stimuli, M1 cells release a high level of pro-inflammatory molecules such as tumor necrosis factor alpha (TNF-α), IL-6, IL-1β, and IL-12 [12]. Furthermore, intracellular markers such as ROS and NO or inducible nitric oxide synthase (iNOS) are also elevated in M1 cells [6] and, therefore, are also considered as useful M1 markers. Besides these intracellular markers and the production of pro-inflammatory factors, M1 cells express on their surface a set of various proteins, including CD80 and CD86 [12].

In contrast to classically activated macrophages, alternatively activated M2 cells reveal anti-inflammatory properties [13]. In general, the M2 subpopulation develops in the presence of transforming growth factor-beta (TGF-β), IL-10, IL-4, and/or IL-13, and releases anti-inflammatory molecules such as IL-4, IL-10, and IL-13 [10]. M2-like cells are characterized by increased expression of intracellular markers such as arginase-1 (Arg-1), and overexpression of surface molecules such as CD163 and CD206 [6,14]. Studies have shown that M2 macrophages can be further subdivided into four subsets, including M2a induced by IL-4 and IL-13, M2b induced by immunocomplexes and ligands of TLRs, M2c induced by IL-10 and glucocorticoids, and M2d induced by IL-6 [14,15]. To date, the role of FRH and interaction between HSPs and TLR-4 in the modulation of macrophage response remain poorly understood [16]. Furthermore, which phenotype of macrophages is induced by FRH, and whether TLR-4 is involved in this process, has not been determined so far. Since changes in macrophage polarization modify the functional activity of these cells, the aim of this study was to identify which phenotype is induced in response to FRH.

We investigated two cell lines of murine macrophages treated with FRH, in which the expression of functional surface markers as well as intracellular markers and cytokines was measured. Our results showed that although FRH-treated macrophages display M2 cell markers, they express proteins involved in pro-inflammatory response such as cyclooxygenase 2 (COX-2) and TLR-4. Furthermore, in response to heat treatment, macrophages are able to produce both pro-inflammatory and anti-inflammatory cytokines. Thus, our data indicate an M2b-like phenotype of heat-treated macrophages.

## 2. Results

### 2.1. Fever-Range Hyperthermia Induces Expression of M2-like Surface Markers

**Figure 1:**
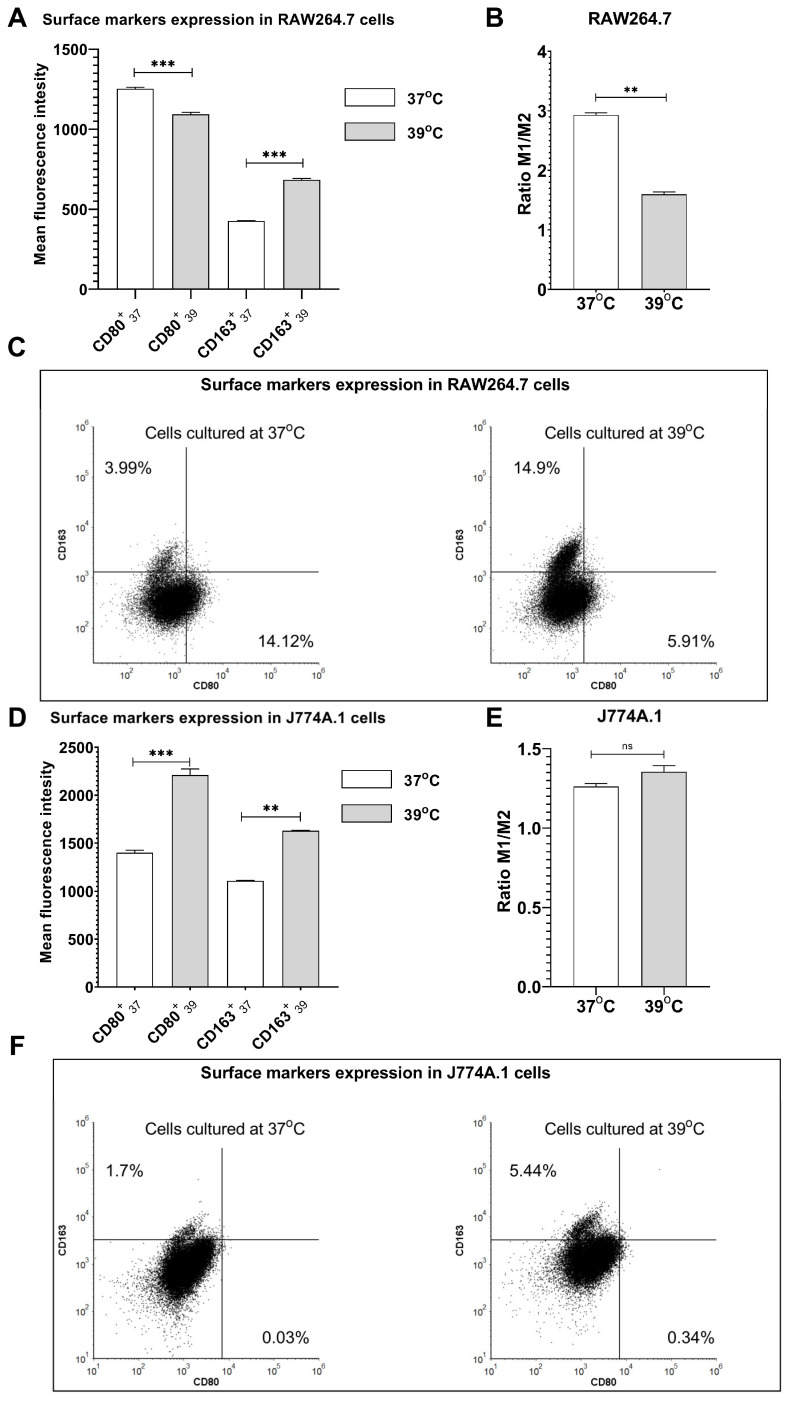
FRH-induced macrophage polarization. RAW264.7 cells (**A**–**C**) and J774A.1 cells (**D**–**F**) were cultured at 37 °C or 39 °C for 24 h. Shadowed bars indicate cells cultured at 39 °C. The expression of surface markers CD80 and CD163 was assessed by flow cytometry. Anti-CD163 antibodies were conjugated with APC, whereas anti-CD80 antibodies were conjugated with FITC. Bars represent the ratio of M1/M2 surface markers (**B**,**E**). Asterisks indicate the statistical significance (ns > 0.05; ** *p* < 0.01; *** *p* < 0.001).

To investigate the ability of FRH to induce macrophage polarization, we analyzed two surface markers: CD80 and CD163. It is known that seeding density significantly affects the expression of surface markers and the release of various cytokines in numerous macrophage cells due to the regulation of intracellular signals that impact the inflammatory response [17,18]. Therefore, in our experiments, all cells were cultured at the same density to limit this effect to spontaneous polarization of macrophages. We observed an elevated number of cells expressing surface marker CD80 in comparison to CD163 in control RAW264.7 cells cultured at 37 °C (Figure 1C). In contrast, FRH treatment upregulated expression of CD163 and decreased the level of CD80 compared to control cells (Figure 1A; *p* < 0.001 and *p* < 0.001, respectively), leading to the increased number of M2-like cells after FRH treatment. Consequently, we observed a substantial decrease in the ratio of M1/M2 surface markers in response to FRH treatment (Figure 1B; *p* < 0.01). Analysis of the expression levels of surface molecules in J774A.1 control cells (cultured at 37 °C) revealed an elevated number of cells expressing CD163 in comparison to CD80 (Figure 1F). The levels of CD163 and CD80 surface markers were potentiated after raising the temperature to the range of 39 °C (Figure 1D; *p* < 0.01, and *p* < 0.001, respectively). However, these changes did not affect the ratio of M1/M2 surface markers in J774A.1 cells (*p* = 0.1425). Collectively, these results indicate the potential of FRH to induce macrophage polarization into M2-like cells more than into M1-like cells.

### 2.2. Fever-Range Hyperthermia Modifies LPS-Induced M1 Polarization towards M2 Phenotype

**Figure 2:**
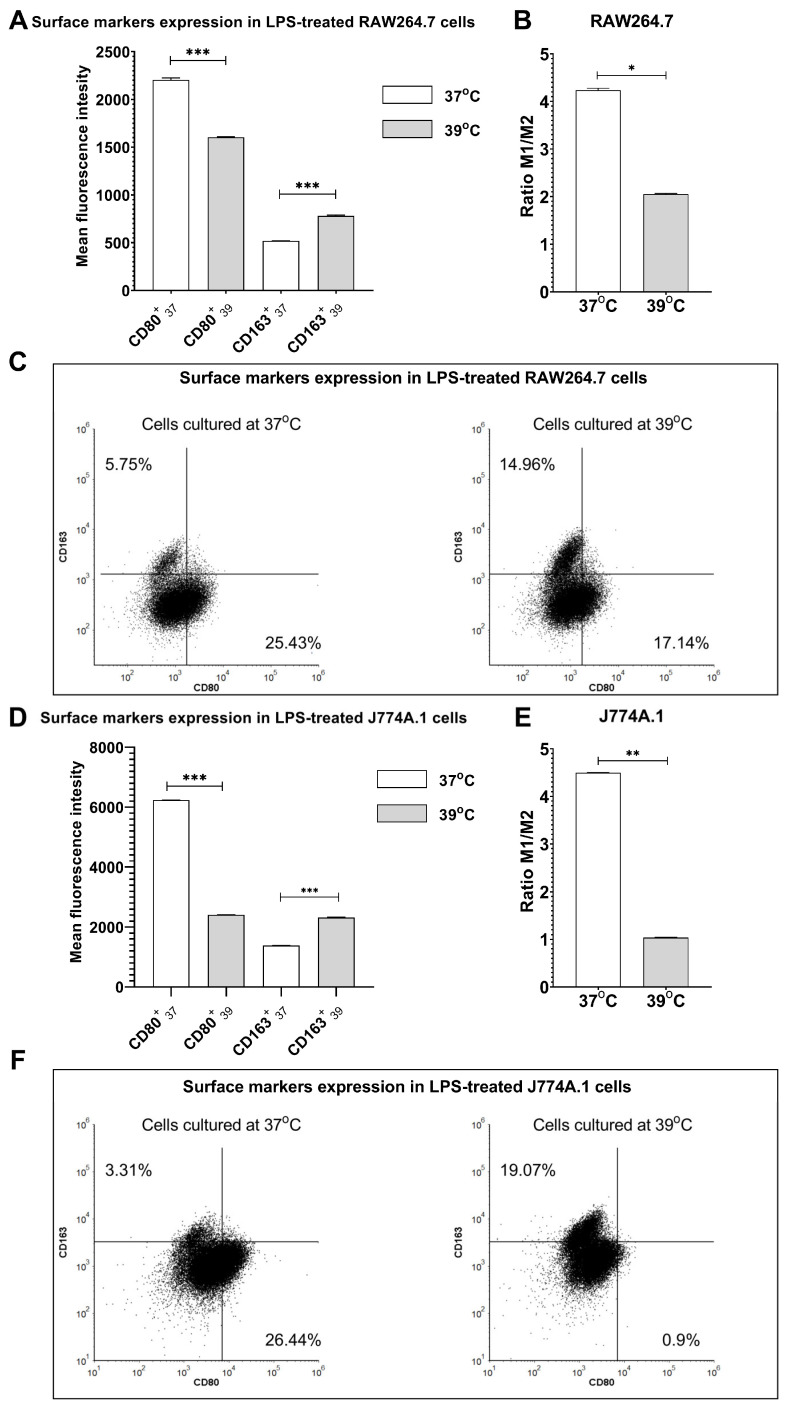
LPS-induced macrophage polarization changes under hyperthermic conditions. RAW264.7 cells (**A**–**C**) and J774A.1 cells (**D**–**F**) were treated with LPS and cultured at 37 °C or 39 °C for 24 h. Shadowed bars indicate cells cultured at 39 °C. The expression of surface markers CD80 and CD163 was assessed by flow cytometry. Anti-CD163 antibodies were conjugated with APC, whereas anti-CD80 antibodies were conjugated with FITC. Bars represent the ratio of M1/M2 surface markers (**B**,**E**). Asterisks indicate statistical significance (* *p* < 0.05; ** *p* < 0.01; *** *p* < 0.001).

To verify the potential of FRH to shift macrophage phenotype in pro-inflammatory conditions, we used LPS, which is also a well-known factor inducing the M1 phenotype. As expected, in LPS-treated RAW264.7 cells and J774A.1 cells cultured at 37 °C, we observed an overexpression of CD80 in comparison to CD163 (Figure 2C,F). Interestingly, we noticed an increased expression of CD163 and decreased expression of CD80 in both LPS-treated cell lines cultured at 39 °C in comparison to cells cultured at 37 °C (Figure 2A,D; *p* < 0.001 in all examined groups). The ratio of CD80/CD163 surface markers was diminished after FRH-treatment in both cell lines (Figure 2B,E; *p* < 0.05 for RAW264.7 cells, and *p* < 0.01 for J774A.1 cells, respectively), which suggests that FRH is able to switch macrophage phenotype into M2-like cells even in pro-inflammatory conditions.

**Figure 3:**
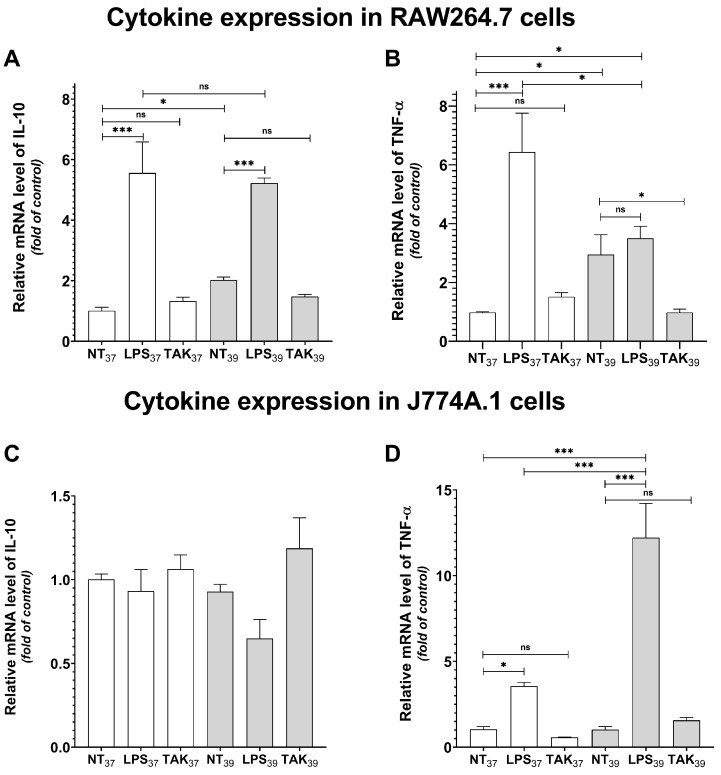
mRNA expression of IL-10 (**A**,**C**) and TNF-α (**B**,**D**) in RAW264.7 cells (**A**,**B**) and J774A.1 cells (**C**,**D**) pre-treated with TAK-242 for 1 h at 37 °C and further treated with LPS at the concentration of 100 ng/mL and cultured at 37 °C and 39 °C for 4 h. Shadowed bars indicate cells cultured at 39 °C. mRNA expression was determined by quantitative real-time PCR. Data represent the mean and standard error of the mean (SEM) obtained from three independent experiments. Asterisks indicate the statistical significance (ns > 0.05; * *p* < 0.05; *** *p* < 0.001).

To expand our investigation into FRH-induced macrophage polarization, we conducted real-time PCR analysis targeting both pro-inflammatory and anti-inflammatory cytokines, which serve as additional markers for assessing the macrophage polarization state. We observed a significant increase in IL-10 expression following heat treatment in RAW264.7 cells (Figure 3A; *p* < 0.05), supporting our earlier findings that FRH induces an M2-like phenotype. Interestingly, we also noted a rise in the mRNA levels of TNF-α after FRH exposure (Figure 3B; *p* < 0.05), suggesting that these cells may exhibit some pro-inflammatory properties. Noteworthily, we detected elevated levels of both cytokines following LPS administration at 37 °C (*p* < 0.001 and *p* < 0.001, respectively). However, upon subsequent heat treatment, we observed no change in the anti-inflammatory IL-10 mRNA levels, but a significant decrease in pro-inflammatory TNF-α (*p* < 0.05). These findings suggest that additional heat treatment in the presence of LPS may mitigate the pro-inflammatory response in RAW264.7 cells. Notably, in our investigations, no statistically significant alterations in anti-inflammatory IL-10 levels were determined in J774A.1 cells (Figure 3C). FRH alone did not affect the level of pro-inflammatory TNF-α. In contrast, we observed a notable upsurge in TNF-α mRNA levels following LPS administration (Figure 3D) (*p* < 0.05), and this effect was markedly potentiated by the supplementary application of heat treatment (*p* < 0.001).

Furthermore, our observations disclosed a noteworthy decrease in TNF-α expression induced by hyperthermia (*p* < 0.05) in RAW264.7 cells following TLR-4 inhibition. This finding suggests that the heat-induced expression of pro-inflammatory cytokines might be reliant on the TLR-4 pathway in RAW264.7 cells, whereas such changes were not evident in J774A.1 cells.

### 2.3. FRH-Induced M2-like Cells Express Pro-Inflammatory Proteins but Reveal Anti-Inflammatory Properties

**Figure 4:**
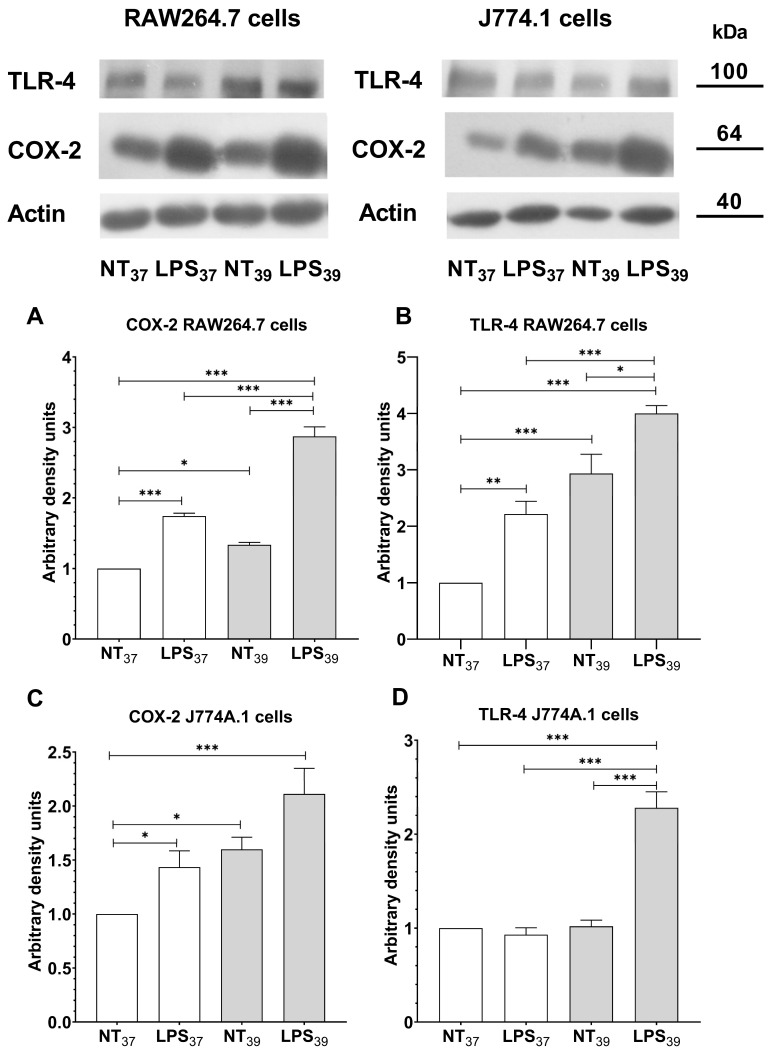
Western blot analysis of cyclooxygenase-2 (COX-2) and Toll-like receptor 4 (TLR-4) expression in RAW264.7 (**A**,**B**) and J774A.1 (**C**,**D**) cells simultaneously treated with LPS and FRH for 2 h. Actin was used as a protein loading control. Data represent the mean and standard error of the mean (SEM) obtained from three independent experiments. Asterisks indicate the statistical significance (* *p* < 0.05; ** *p* < 0.01; *** *p* < 0.001).

Since we observed that FRH changes the phenotype of macrophages into M2-like cells, we wanted to verify whether this may influence the expression of key molecules involved in inflammatory response, such as TLR-4 and COX-2. As expected, we observed an increased expression of COX-2 and TLR-4 in RAW264.7 cells treated with LPS (Figure 4A,B; *p* < 0.001 and *p* < 0.01, respectively). Interestingly, FRH alone also induced upregulated expression of both COX-2 and TLR-4 in RAW264.7 cells in comparison to control cells (*p* < 0.05, and *p* < 0.001, respectively). Furthermore, we observed that the co-treatment with FRH and LPS had an additive effect on the increased expression of both examined proteins in RAW264.7 cells (*p* < 0.001 for both COX-2 and TLR-4). Similarly to the RAW264.7 cell treatment, we observed increased expression of COX-2 in J774A.1 cells stimulated with LPS at 37 °C (Figure 4C; *p* < 0.05). Of note, FRH alone induced upregulated COX-2 expression in J774A.1 cells (*p* < 0.05), and this effect was even higher in cells simultaneously treated with LPS and FRH (*p* < 0.001). We did not notice any changes in TLR-4 expression induced by LPS at 37 °C or under the influence of FRH itself in J774A.1 cells (Figure 4D). However, co-treatment with LPS and FRH induced a significant increase in TLR-4 expression in J774A.1 cells (*p* < 0.001).

**Figure 5:**
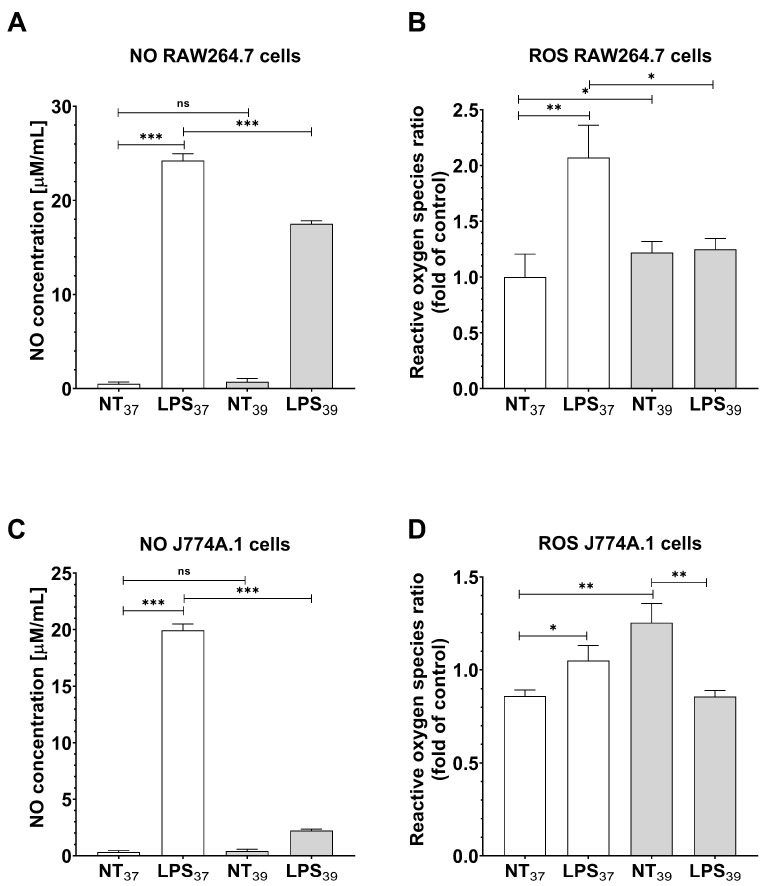
Oxidative status of RAW264.7 (**A**,**B**) and J774A.1 (**C**,**D**) cells in response to simultaneous treatment with FRH and LPS for 24 h. The effect was measured as NO concentration (colorimetric) and the relative level of ROS (fluorescent) assessed by flow cytometry. Data represent the mean and standard error of the mean (SEM) obtained from three independent experiments. Asterisks indicate the statistical significance (ns *p* > 0.05; * *p* < 0.05; ** *p* < 0.01; *** *p* < 0.001).

Since we observed increased expression of surface markers specific to M2-like cells and increased expression of pro-inflammatory TNF-α, we wanted to check the influence of FRH on the functional activity of macrophages. Therefore, to verify whether FRH may influence the response of cells to infection, we examined the level of ROS and NO. We confirmed a commonly known fact that in RAW264.7 and J774.1 cells, treatment with LPS increases levels of NO and ROS (Figure 5A,B; *p* < 0.001 and *p* < 0.01, respectively, and Figure 5C,D; *p* < 0.001 and *p* < 0.05, respectively). This effect was abolished by additional treatment of RAW264.7 cells with FRH (*p* < 0.001 and *p* < 0.05, respectively). Furthermore, FRH alone did not induce the production of NO in RAW264.7 cells. However, FRH induced a slight increase in ROS level in comparison to control cells (*p* < 0.05).

In J774.1 cells, we observed that FRH alone did not affect the NO level, whereas ROS production was increased (*p* < 0.01). Furthermore, similarly to RAW264.7 cell treatment, we observed a significant decrease in NO and ROS levels in J774A.1 cells simultaneously treated with LPS and FRH in comparison to LPS alone (*p* < 0.001 and *p* < 0.05, respectively).

### 2.4. Fever-Range Hyperthermia Changes Macrophage Phenotype in a TLR-4-Independent Way?

**Figure 6:**
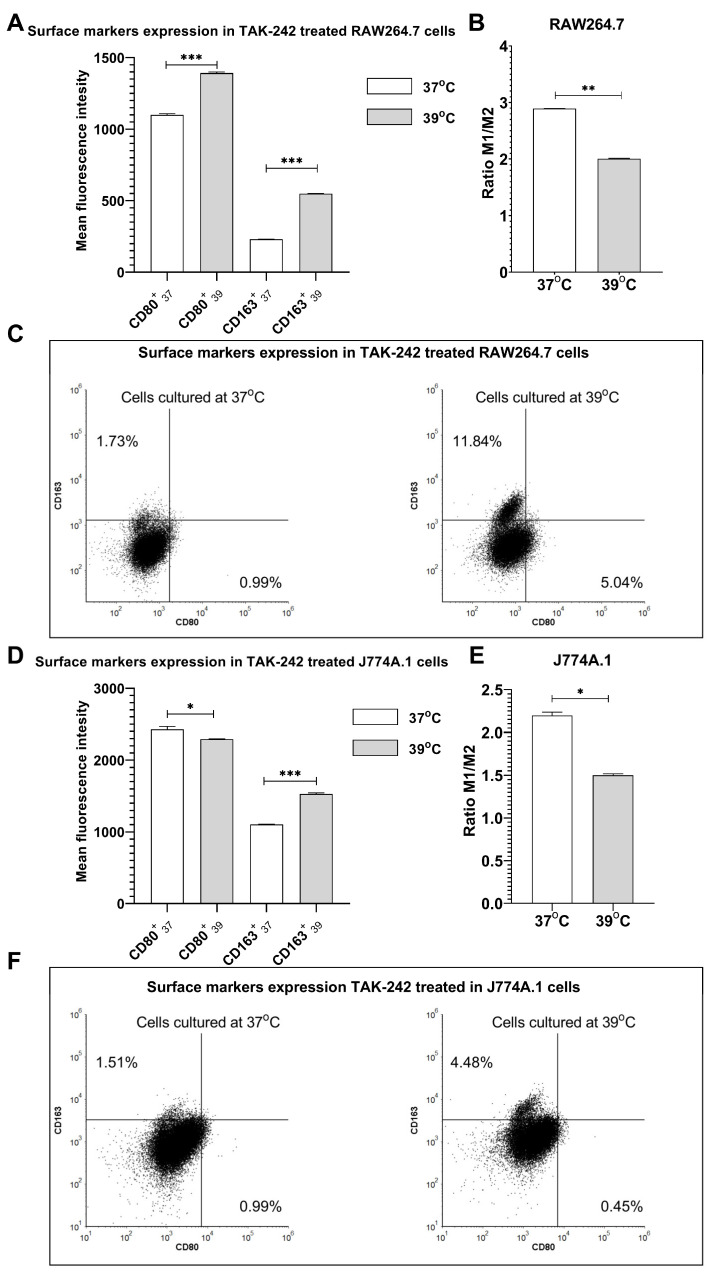
Macrophage polarization surface markers after TLR-4 inhibition. RAW264.7 cells (**A**–**C**) and J774.A cells (**D**–**F**) were pre-treated with 0.1 µM TAK-242 for 1 h at 37 °C, and further cultured at 37 °C and 39 °C for 24 h. Shadowed bars indicate cells cultured at 39 °C. The expression of surface markers CD80 and CD163 was assessed by flow cytometry. Anti-CD163 antibodies were conjugated with APC, whereas anti-CD80 antibodies were conjugated with FITC. Bars represent the ratio of M1/M2 surface markers (**B**,**E**). Asterisks indicate statistical significance (* *p* < 0.05; ** *p* < 0.01; *** *p* < 0.001).

It is well known that in response to hyperthermia, various heat shock proteins, including HSP-70, are released in RAW264.7 cells. Similarly, it has been proved that HSPs can activate the TLR-4 downstream signaling pathway [9]. Since TLR-4 is an important receptor involved in macrophage polarization into both phenotypes [6], we wondered whether inhibition of TLR-4 might affect FRH-induced macrophage polarization. We employed TAK-242, a small-molecule-specific inhibitor of the TLR-4 signaling pathway [19]. We observed that inhibition of TLR-4 decreased the spontaneous polarization of non-treated RAW264.7 cells into the M1 phenotype, which was previously presented in Figure 1 (Figure 6C). Interestingly, after additional treatment with FRH we observed overexpression of both surface markers CD163 and CD80 (Figure 6A; *p* < 0.001 and *p* < 0.001, respectively) in comparison to cells cultured at 37 °C. However, we observed a decrease in the ratio of M1/M2 surface markers after FRH treatment in RAW264.7 cells (Figure 6B; *p* < 0.01). We did not discern substantial variations in the surface marker expression of J774A.1 cells cultured at 37 °C upon the inhibition of TLR-4, as compared to the outcomes illustrated in Figure 1 (Figure 6F). However, following the supplementary heat treatment, we noted a remarkable reduction in CD80 expression and a statistically significant elevation in the CD163 surface marker levels in J774A.1 cells (Figure 6D; *p* < 0.05, and *p* < 0.001, respectively). Consequently, a noteworthy decrease in the M1/M2 surface marker ratio was observed (Figure 6E; *p* < 0.05). Thus, we hypothesized that FRH-induced polarization into M2-like cells, measured as surface marker expression was a TLR-4-independent phenomenon. 

**Figure 7:**
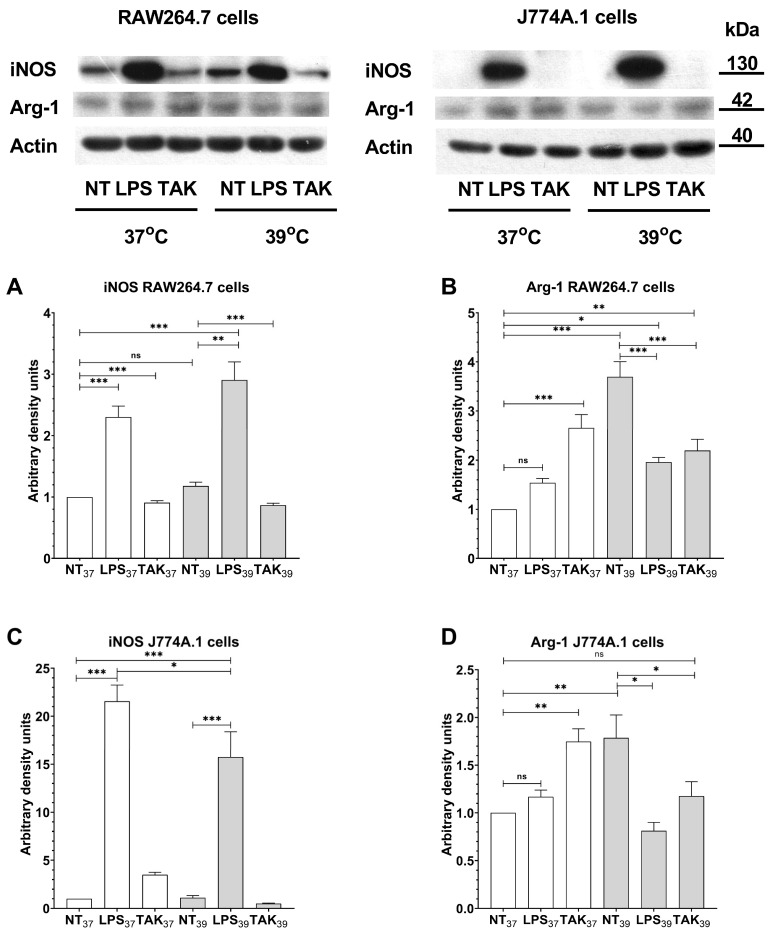
Western blot analysis of inducible nitric oxide synthase (iNOS) and Arginase-1 (Arg-1) in RAW264.7 cells (**A**,**B**) and J774.A cells (**C**,**D**) pre-treated with TAK-242 for 1 h at 37 °C, and further cultured at 37 °C and 39 °C for 24 h. Actin was used as a protein loading control. Data represent the mean and standard error of the mean (SEM) obtained from three independent experiments. Asterisks indicate the statistical significance (ns *p* > 0.05; * *p* < 0.05; ** *p* < 0.01; *** *p* < 0.001).

Next, to extend our research on FRH-induced macrophage polarization, we analyzed intracellular markers such as inducible Nitric Oxide Synthase (iNOS) and Arginase-1 (Arg-1). As we expected, LPS increased the expression of iNOS, (a marker of M1 cells) in RAW264.7 cells (Figure 7A; *p* < 0.001), and TLR-4 inhibition did not affect this expression in RAW264.7 cells in both thermal conditions (*p* < 0.001, and *p* < 0.001, respectively). We also detected a slight increase in iNOS expression after FRH alone; however, this change was statistically insignificant (*p* = 0.94). Interestingly, simultaneous treatment with LPS and FRH triggered upregulated iNOS expression in comparison to control cells (*p* < 0.001). 

In J774A.1 cells, we noticed overexpression of iNOS after LPS administration in both examined temperatures (Figure 7C; *p* < 0.001 and *p* < 0.001, respectively). Similarly to RAW264.7, we did not observe changes in iNOS level after FRH alone. However, simultaneous treatment of J774A.1 cells with LPS and FRH reduced the level of iNOS in comparison to LPS alone (*p* < 0.05).

In the case of the measurement of Arg-1 (a marker of M2 cells), we noticed a statistically insignificant increase in Arg-1 expression in both examined cell lines RAW264.7 and J774A.1 after LPS treatment at 37 °C (Figure 7B,D). Surprisingly, in RAW64.7 cells, the Arg-1 level was upregulated after inhibition of TLR-4 by TAK-242 in both thermal conditions in comparison to the control group (*p* < 0.001 and *p* < 0.01, respectively), whereas in J774A.1 cells, a significant increase was observed only at 37 °C (*p* < 0.01). In accordance with surface markers, we observed a significant increase in Arg-1 after FRH treatment in both tested cell lines (*p* < 0.001 for RAW264.7 cells and *p* < 0.01 for J774A.1 cells). In both cell lines, this effect was abolished by additional treatment with LPS at 39 °C (*p* < 0.001 for RAW264.7 and *p* < 0.05 for J774A.1). However, in RAW264.7 cells, the level of Arg-1 was still upregulated in comparison to non-treated cells at 37 °C (*p* < 0.05).

## 3. Discussion

Fever-range hyperthermia (FRH) is a condition used in research to investigate a thermal component of fever [1,20]. FRH is also a medical procedure that may trigger benefits in patients who suffer from chronic diseases such as rheumatic diseases and some cardiovascular disorders [3]. Additionally, FRH can support standard cancer treatments, including chemotherapy and radiation [21]. Although it is known that macrophages are sensitive to heat [4], relatively little attention has been given to the underlying molecular mechanism.

Macrophages are cells that are recognized as the early warning system, swiftly sounding the alarm in response to infection [22]. To protect the body against infections, macrophages produce various molecules, including reactive oxygen species (ROS) and nitrogen oxide (NO) [23,24]. Of note, ROS and NO are commonly known markers of the M1 phenotype in macrophages [23]. Both of these molecules induce oxidative stress and activate inflammation. Dysregulation of these molecules can lead to chronic inflammation and tissue damage, and therefore, it is important to identify factors that can modulate their level in a body [24]. In our research, we observed that FRH alone did not induce oxidative stress in macrophages, which is in accordance with results published by others [5,25]. Furthermore, in LPS-treated cells, which produce increased levels of ROS and NO [26,27], we observed that FRH was able to diminish this effect. 

Macrophages represent a continuum of highly plastic effector cells, resembling a spectrum of diverse phenotype states. Depending on their phenotype, macrophages can play either a pro-inflammatory or anti-inflammatory role [6]. Whether the heat (e.g., produced during fever or during therapy with FRH) can affect this process has not been determined yet. We revealed that FRH alone induces an M2-like phenotype manifested by increased expression of CD163, IL-10, and Arg-1. However, we observed an increased level of pro-inflammatory TNF-α, which was in line with our previous experiments showing that FRH-treated macrophages can release pro-inflammatory cytokines such as IL-6 and IL-β [4]. Other authors have found that stimulation of RAW264.7 cells induces a heightened responsiveness to lipopolysaccharide in a model of inflammatory responses when compared to J774A.1 macrophages [28]. Consistent with these findings, we noted analogous discrepancies in cytokine expression patterns following both LPS and FRH treatments. In accordance, other studies showed that FRH may induce overexpression of pro-inflammatory cytokines, including TNF-α and IL-6 [25,29]. Although research seemingly contradicted the general M1/M2 polarization, we managed to classify these cells using additional classifications among M2-like cells [30,31]. Thus, our data indicate that FRH induces an M2-like phenotype, and it is highly probable that these cells belong to the M2b subtype. M2b macrophages are considered as regulatory cells [30] because, except for the pro-inflammatory cytokines such as IL-1β, TNF-α or IL-6, these macrophages produce a high amount of anti-inflammatory IL-10 [6]. It is believed that M2b macrophages are effective at suppressing inflammation in a process that is IL-10 dependent [6,30]. 

The expression of COX-2 in various tissues is stimulated by pro-inflammatory molecules like IL-6, IL-1β, and TNF-α. As a crucial enzyme in fever induction, cyclooxygenase 2 catalyzes the synthesis of prostaglandins, particularly prostaglandin E2 (PGE2). PGE2 acts on the hypothalamus, resetting the body’s temperature set-point, thereby promoting fever [32,33]. In our studies, we noted a substantial increase in COX-2 expression triggered by FRH. In accordance with our results, other authors observed that increased expression of COX-2 may induce polarization of macrophages into the M2 phenotype through the PGE2–EP4 axis [34]. This suggests that not only FRH, but also COX-2-dependent fever are regulators of macrophage polarization.

Generally, it is believed that Toll-like receptors, particularly the TLR-4 signaling pathway activated by LPS, polarize macrophages towards the M1 cells [35]**.** Indeed, we observed that LPS alone induces a shift into the M1 phenotype in macrophages cultured at 37 °C. This was manifested by increased expression of M1 surface marker CD80, and increased levels of intracellular markers such as ROS, NO, and inducible nitric oxidase. However, we found that FRH significantly affects polarization of macrophages triggered by LPS and, in consequence, M1 macrophages start to express M2 markers. Furthermore, we noticed that FRH treatment decreased the LPS-induced level of TNF-α in the RAW264.7 cell line. Comparatively to our findings, Ostberg et al. (2000) showed that FRH in vitro decreases the level of LPS-induced pro-inflammatory cytokines such as IL-6, TNF-α and IL-1β in cells isolated from the peritoneal cavity [36].

There are studies showing that in the response to heat treatment, heat shock proteins are released [9,37]. These proteins have been identified as molecules that interact with TLR-4 [9]. Furthermore, classical M1 macrophages exhibit lower levels of TLR-2 and TLR-4 expression when compared to alternatively activated M2 macrophages [38]. The reduction in TLR-4 expression within M1 macrophages may serve as a protective mechanism aimed at preventing the onset of an exacerbated immune response [39]. These results align with our own findings, which demonstrated an elevated TLR-4 expression in M2-like cells following FRH treatment. Therefore, we wondered whether TLR-4 is involved in FRH-induced macrophage polarization. We used TAK-242, an inhibitor of TLR-4 [19] that prevents activation and downstream signaling, leading to the inhibition of the inflammatory response. Published data showed the potential of TAK-242 to induce M2 polarization in microglial cells [40], which is in line with our results. Since it is believed that FRH acts through the release of HSPs and activation of the TLR-4 pathway [9,41], we wanted to check whether this pathway is involved in macrophage polarization. The inhibition of TLR-4 through the use of TAK-242 is established in the literature to reduce the levels of proinflammatory cytokines, including IL-6 and TNF-α, in response to stimuli such as LPS [42]. In the current study, the inhibition of TLR-4 similarly led to a suppression of FRH-induced TNF-α expression. Additionally, the partial inhibition of FRH-induced Arg-1 expression by TAK-242 suggests a regulatory impact on the M2-like polarization of macrophages. However, the unaffected expression of surface marker CD163 and IL-10 in the presence of TAK-242 indicates that TLR-4 inhibition may not significantly influence these markers associated with macrophage polarization. 

Overall, these findings suggest that TLR-4 plays an important role in modulating specific aspects of macrophage polarization, particularly in the context of cytokine expression. 

In conclusion, our research showed that FRH may significantly diminish macrophage-induced acute inflammatory response and thus could be used in therapies that require anti-inflammatory action. Furthermore, since FRH may be considered as a model to investigate the thermal component of fever, it is likely that a febrile increase in body temperature regulates macrophage polarization by inducing the M2b phenotype. It is known, that the differentiation of macrophages into such a regulatory phenotype contributes to the resolution of inflammation [43]. Thus, it seems that fever-associated heat may be an important regulator that shifts macrophages from the pro-inflammatory M1 phenotype that develops at the beginning of infection towards regulatory M2b, to enhance tissue repair and regeneration. It is likely that inhibition of fever keeps macrophages in the pro-inflammatory M1 phenotype and may lead to harmful effects. This issue, however, needs further research.

## 4. Materials and Methods

### 4.1. Cell Culture

The murine macrophage RAW264.7 cell line was obtained from the European Collection of Cell Cultures (cat. No. 91062702; Salisbury, UK), and the murine macrophage J774A.1 cell line was a gift from Prof. Katarzyna Kwiatkowska, PhD, DSc, of the Nencki Institute of Experimental Biology of the Polish Academy of Sciences. The RAW264.7 and J774A.1 cells were cultured in high- or low-glucose DMEM culture medium, respectively (Biowest; Nuaillé, France). Both culture media were supplemented with 10% heat-inactivated fetal bovine serum (FBS) (Sigma Aldrich, Darmstadt, Germany) and a mixture of antibiotics (100 µg/mL streptomycin and 100 IU/mL penicillin) (Sigma Aldrich). Cells were maintained under controlled conditions at 37 °C in a humidified atmosphere with 5% CO_2_. Depending on cell confluence, the culture media were changed every 2 or 3 days. In order to collect the cells, they were rinsed with fresh media, and cell scrapers were used to remove adherent cells from the culture flasks.

### 4.2. Measurement of Intracellular Reactive Oxygen Species (ROS)

The effects of co-treatment with heat and LPS derived from *Escherichia coli* 0111:B4 (Sigma-Aldrich) on ROS generation in TLR-4 inhibited and non-inhibited RAW264.7 and J774A.1 cells were measured by H_2_DCF_DA (Sigma-Aldrich) staining, followed by flow cytometry analysis as described previously [4]. Cells were seeded in 6-well plates at the density of 1 × 10^6^ cells per well. Following overnight pre-incubation, a 1 h pretreatment with TAK-242 (TLR-4 inhibitor purchased from Cayman Chemical, Ann Arbor, MI, USA) at a concentration of 0.1 µM at 37 °C was carried out. Next, LPS at a concentration of 100 ng/mL was added, and cells were incubated for 24 h at 37 °C or 39 °C. Then, cells were harvested, washed twice with PBS, and cultured in a serum-free transparent DMEM medium containing 10 μM H_2_DCFH_DA for 30 min at 37 °C in the dark. After incubation with fluorescent dye, cells were washed twice with PBS, and DCF fluorescence distribution was detected by flow cytometry using BriCyte E6 (Mindray, Shenzhen, China) at an excitation wavelength of 488 nm and an emission wavelength of 525 nm. Results are presented as the ratio of a geometric mean of treated/control cells (fold of control).

### 4.3. Nitric Oxide Determination

Nitric oxide evaluation was carried out with the Griess reagent (modified) (Sigma-Aldrich) according to the manufacturer’s instructions. The detection solution was prepared with ultrapure distilled water, and the analysis was conducted in the presence of a standard curve in a range of 0.5–65 µM of NO_2_^−^. RAW264.7 and J774A.1 cells were seeded in the DMEM media without phenol red (Biowest) at the density of 5 × 10^5^ cells/well in a 24-well plate. Following a 1 h pretreatment with TAK-242 at a concentration of 0.1 µM at 37 °C, cells were simultaneously treated with 100 ng/mL LPS and exposed to 37 °C or 39 °C for 24 h. After treatment, the supernatants were collected, centrifuged, and mixed in equal volumes with Griess reagent. The absorbance was read at 540 nm after 15 min using a Synergy HT Multi-Mode microplate reader (BioTek Instruments, Winooski, VT, USA).

### 4.4. Western Blot Analysis

To evaluate TLR-4, COX-2, iNOS, and Arg-1 expression, Western blot analysis was conducted. RAW264.7 and J774A.1 cells were seeded in 24-well plates at the density of 5 × 10^5^ cells/well. Following overnight pre-incubation and a 1 h pretreatment with TAK-242 at a concentration of 0.1 µM at 37 °C [44], 100 ng/mL of LPS was added, and cells were incubated for 2 h or 24 h (depending on the experiment) at 37 °C or 39 °C. After incubation, the cells were rinsed with ice-cold PBS and lysed in 100 µL of a 1 x RIPA buffer supplemented with 1% SDS and 0.5% protease inhibitor cocktail (all reagents were purchased from Sigma Aldrich). Following mechanical homogenization, the lysates underwent centrifugation to eliminate cellular debris. Subsequently, the samples were subjected to heating at 95 degrees Celsius for a duration of 5 min. To evaluate the protein concentration in the lysates, the Pierce™ BCA Protein Assay Kit (Thermo Fischer Scientific, Waltham, MA, USA) was used, according to the manufacturer’s instructions. After dilution of lysates with sample buffer to a final concentration of 1 mg/mL, SDS-PAGE electrophoresis was performed using 20 µL of sample and 4–20% precast polyacrylamide gels (Bio-Rad, Hercules, CA, USA). Following the transfer onto nitrocellulose, the membranes were immunoblotted with appropriate primary antibodies, followed by incubation with secondary antibodies conjugated with horseradish peroxidase (HRP). All antibodies used in these studies are described in Table 1. Immunoreactive bands were visualized by chemiluminescence using SuperSignal West Pico substrate (Thermo Fisher Scientific). The densitometrical analysis was conducted using the ImageJ program 2.1.0/1.53q (National Institute of Mental Health, Bethesda, MD, USA).

### 4.5. Analysis of Macrophage Polarization by Flow Cytometry

Murine macrophage cell lines RAW264.7 and J774A.1 were seeded in 12-well plates at a density of 1 × 10^6^ cells/well. The surface expression of CD80 as a marker of M1-phenotype polarization and CD163 as a marker of M2-phenotype polarization was analyzed. Following 1 h pretreatment with 0.1 µM TAK-242 at 37 °C, the cells were simultaneously exposed to LPS (100 ng/mL) and FRH for 24 h. Then, macrophage polarization was investigated by flow cytometry analysis. Cells were collected into separate tubes and washed twice with a washing buffer containing ice-cold PBS, 1% bovine serum albumin, and 0.02% sodium azide (all reagents were purchased from Sigma-Aldrich). The blocking of Fc receptors was carried out with Mouse Seroblock FcR (Bio-Rad) for 10 min in the dark at 4 °C, according to the manufacturer’s instructions. Then, the cells were incubated with fluorescently labeled antibodies (FITC anti-mouse CD80, APC anti-mouse CD163, both purchased from Sony Biotechnology Inc., San Jose, CA, USA) on ice for an additional 30 min. Next, cells were washed and analyzed by BriCyte E6 flow cytometer. Cells were readily discerned on scatter plots, and debris was eliminated from the analysis. Unstained cells, as well as those solely stained with either anti-CD80 or anti-CD163, were employed as negative controls to assess background fluorescence and establish compensation settings. The data were evaluated in FlowJo v10 software (Becton, Dickinson & Company, Franklin Lakes, NJ, USA) and presented as the mean fluorescence intensity, % highly expressing marker cells, and the CD80/CD163 intensity ratio of expressing cells in the population of 2 × 10^4^ events.

### 4.6. Analysis of Cytokine Expression by Real-Time PCR

For qPCR analysis, both macrophage cell lines were seeded at a density of 1.5 × 10^5^ cells per well in 12-well plates. After an overnight pre-incubation period, the cells underwent a 1 h pretreatment with TAK-242 at a concentration of 0.1 µM at 37 °C, followed by stimulation with LPS (100 ng/mL) for 4 h at 37 °C or 39 °C. Total mRNA extraction was performed using PureZOL™ RNA Isolation Reagent (Bio-Rad), following the manufacturer’s guidelines based on the Chomczynski–Sacchi method [45]. The concentration of RNA in the samples was determined using a Take3 Micro-Volume Plate with the Synergy HT Multi-Mode Microplate Reader (BioTek Instruments). Subsequently, cDNA synthesis was conducted using 1 µg of total RNA and iScript™ Reverse Transcription Supermix for RT-qPCR (Bio-Rad), as per the manufacturer’s instructions. Quantitative real-time PCR was conducted following the manufacturer’s instructions in a final volume of 10 µL. Each reaction mixture comprised cDNA, iTaq Universal SYBR^®^ Green Supermix (Bio-Rad), and the PrimePCR™ SYBR^®^ Green Assay designed for IL-10 (Unique Assay ID: qMmuCED0044967) and TNF-α (Unique Assay ID: qMmuCED0004141) amplification. The amplification was performed using the CFX Connect Real-Time PCR Detection System (Bio-Rad). To ensure data accuracy, the housekeeping gene GAPDH (Unique Assay ID: qMmuCED0027497), also provided by Bio-Rad, was utilized for data normalization. Data analysis was carried out using the double delta Ct analysis method (2^−ΔΔCt^). Additionally, a melt curve analysis was performed during each qPCR run to identify any potential non-specific primer binding. All test samples were run in triplicate. 

### 4.7. Statistical Analysis

All values are reported as mean ± standard error of the mean (SEM) of three independent experiments. Statistical significance was determined using analysis of variance (two-way ANOVA), followed by the Tukey test at a critical value of *p* < 0.05.

## Figures and Tables

**Table 1:** Primary and secondary antibodies used for Western blot analysis.

Primary Antibodies				
Protein Name	Protein Symbol	Cat. No.	Source/Isotype	Company
Cyclooxygenase 2	COX-2	#12282	Rabbit IgG	Cell Signaling Technology (Danvers, MA, USA)
Inducible Nitric Oxide Synthase	iNOS	#2982	Rabbit IgG	Cell Signaling Technology
Toll-like Receptor 4	TLR-4	#14358	Rabbit IgG	Cell Signaling Technology
Arginase-1	Arg-1	#93668	Rabbit IgG	Cell Signaling Technology
Actin	Actb	612657	Mouse IgG	BD Bioscience (Franklin Lakes, NJ, USA)
**Secondary Antibodies**				
**Target**	**Origin**		**Type of conjugate**	**Company**
Anti-Rabbit	Goat IgG		Peroxidase-conjugated Anti-Rabbit	Sigma Aldrich
Anti-Mouse	Goat IgG		Peroxidase-conjugated Anti-Mouse	Jackson ImmunoResearch Laboratories, Inc. (West Grove, PA, USA)

## Data Availability

The original contributions presented in the study are included in the article/Supplementary Materials; further inquiries can be directed to the corresponding author/s.

## Supplementary Materials

The following supporting information can be downloaded at: https://www.mdpi.com/article/10.3390/ijms242417574/s1.
