# Supplementary material for: Fever-Range Hyperthermia Promotes Macrophage Polarization towards Regulatory Phenotype M2b
Source: Int J Mol Sci. 2023 Dec 17;24(24):17574. doi: 10.3390/ijms242417574 (PMC10744093; doi:10.3390/ijms242417574)
Supplement: Supplementary file 1 [file ijms-24-17574-s001.zip › ijms-2753399-supplementary.pdf]

Full unedited blot for figure 3

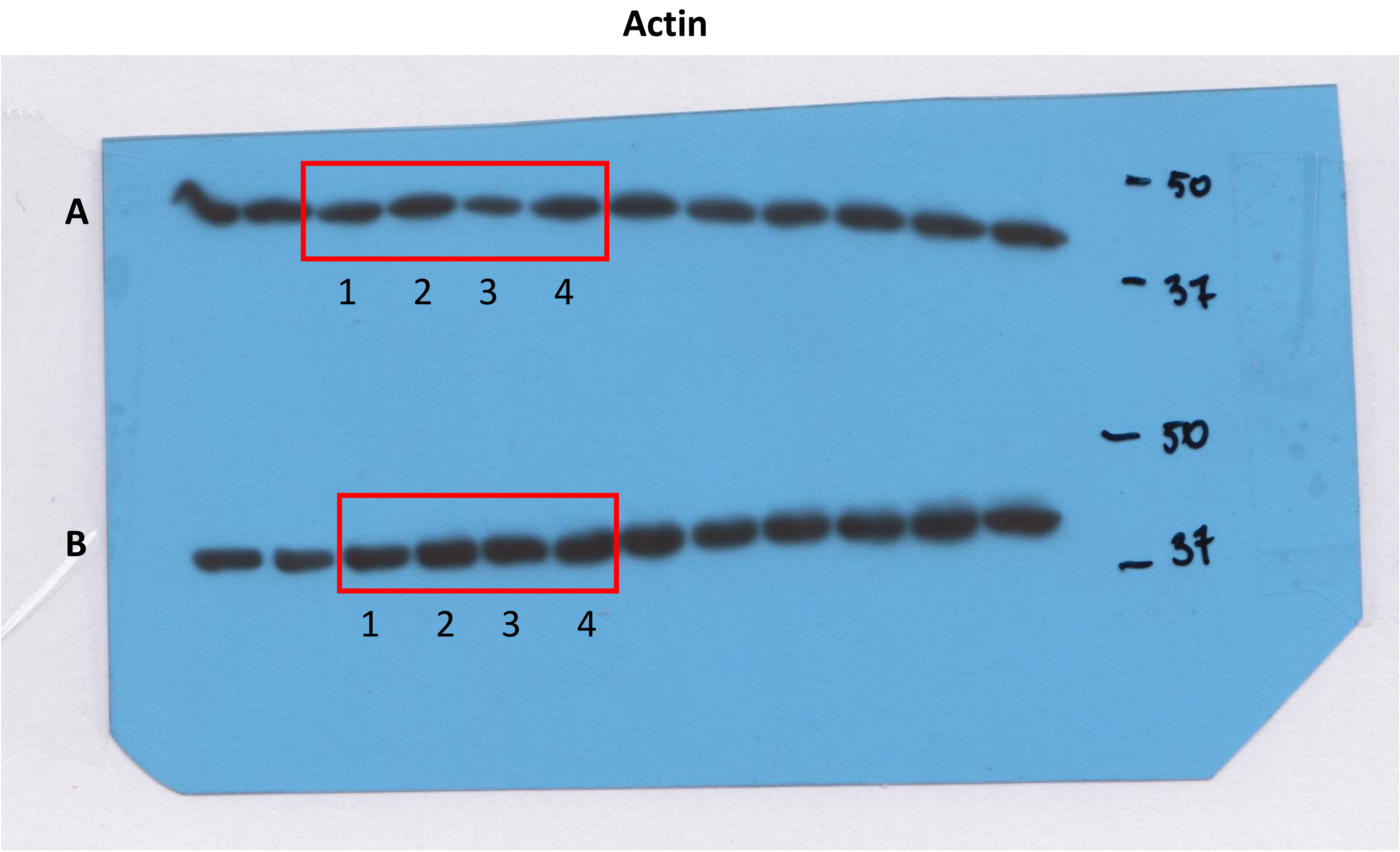

1. NT_{37}
2. LPS_{37}
3. NT_{39}
4. LPS_{39}

A) J774.1A cells
B) RAW264.7 cells

Full unedited blot for figure 3

COX-2

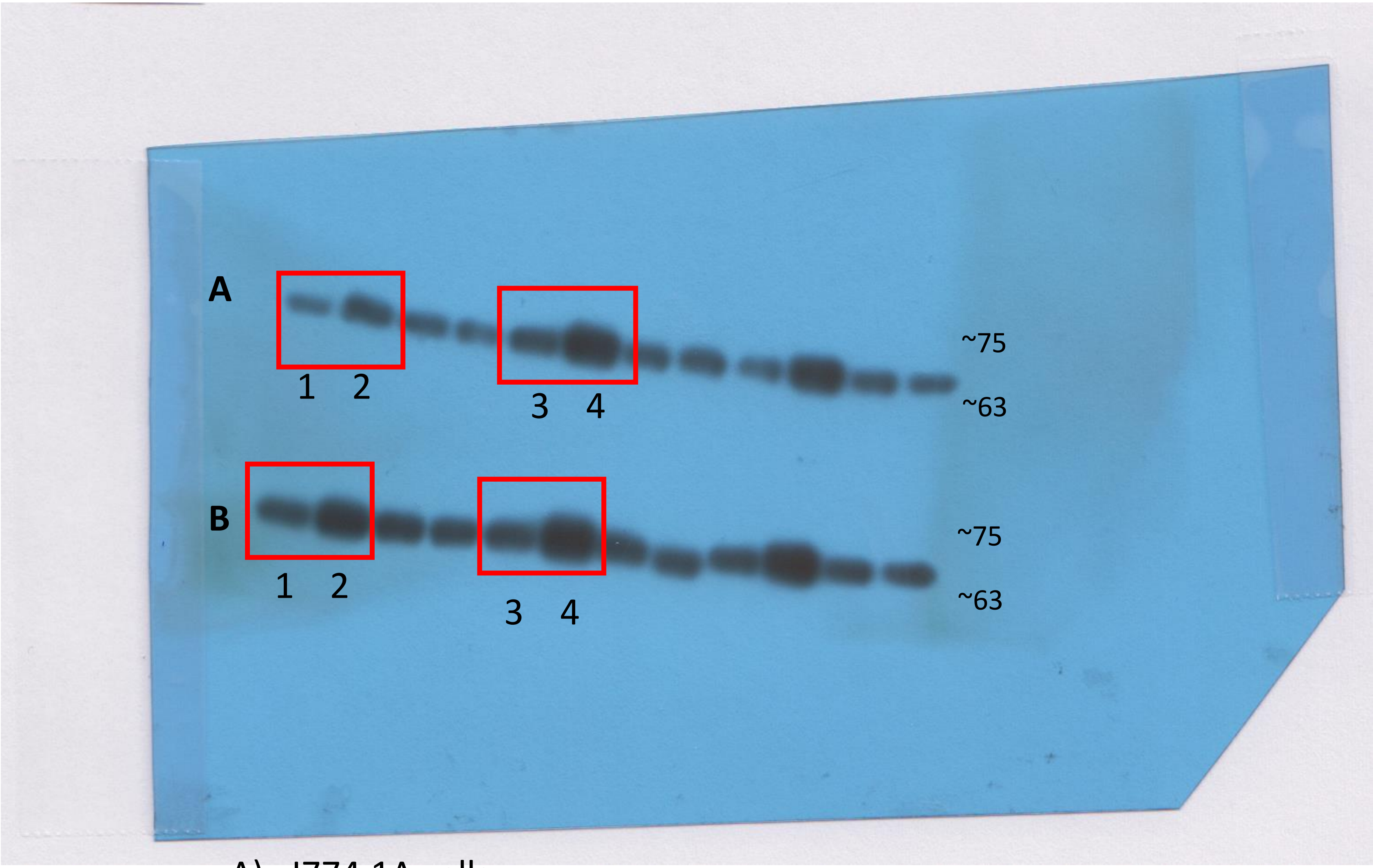

1. NT_{37}
2. LPS_{37}
3. NT_{39}
4. LPS_{39}

A) J774.1A cells
B) RAW264.7 cells

Full unedited blot for figure 3

TLR-4

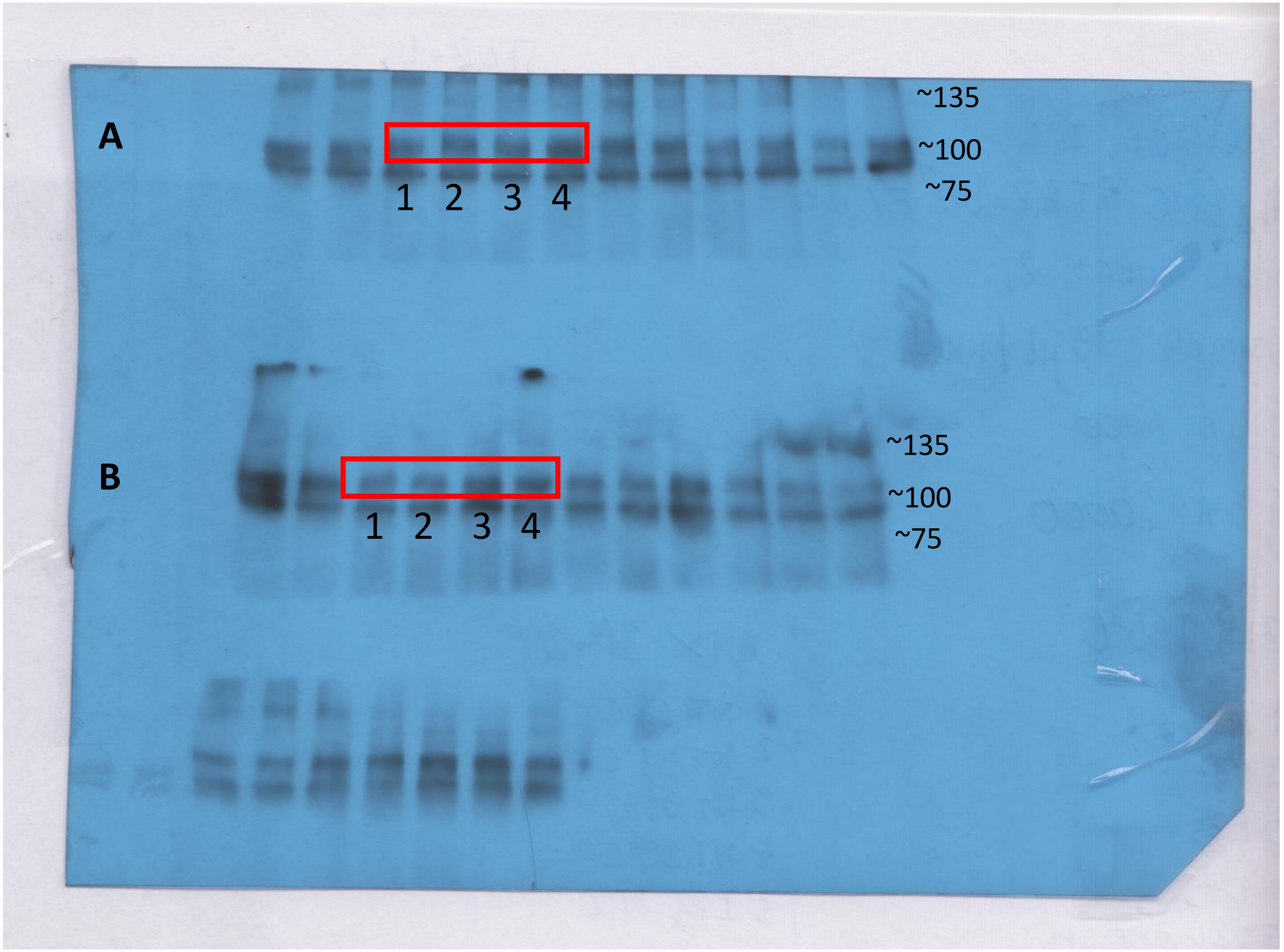

1. NT_{37}
2. LPS_{37}
3. NT_{39}
4. LPS_{39}

A) J774.1A cells
B) RAW264.7 cells

Full unedited blot for figure 6

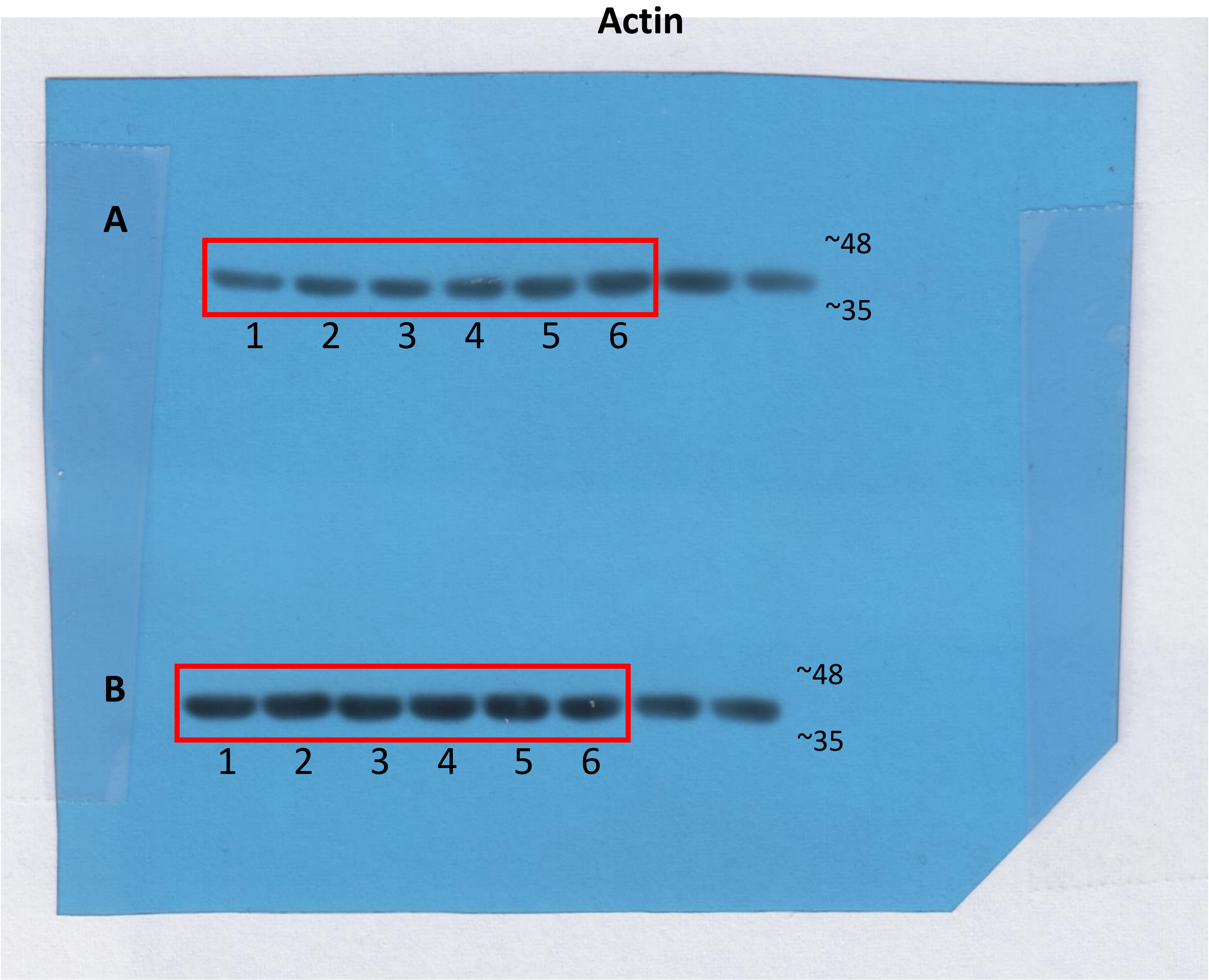

A) J774.1A cells
B) RAW264.7 cells

1. NT_{37}
2. LPS_{37}
3. TAK_{37}
4. NT_{39}
5. LPS_{39}
6. TAK_{37}

Full unedited blot for figure 6

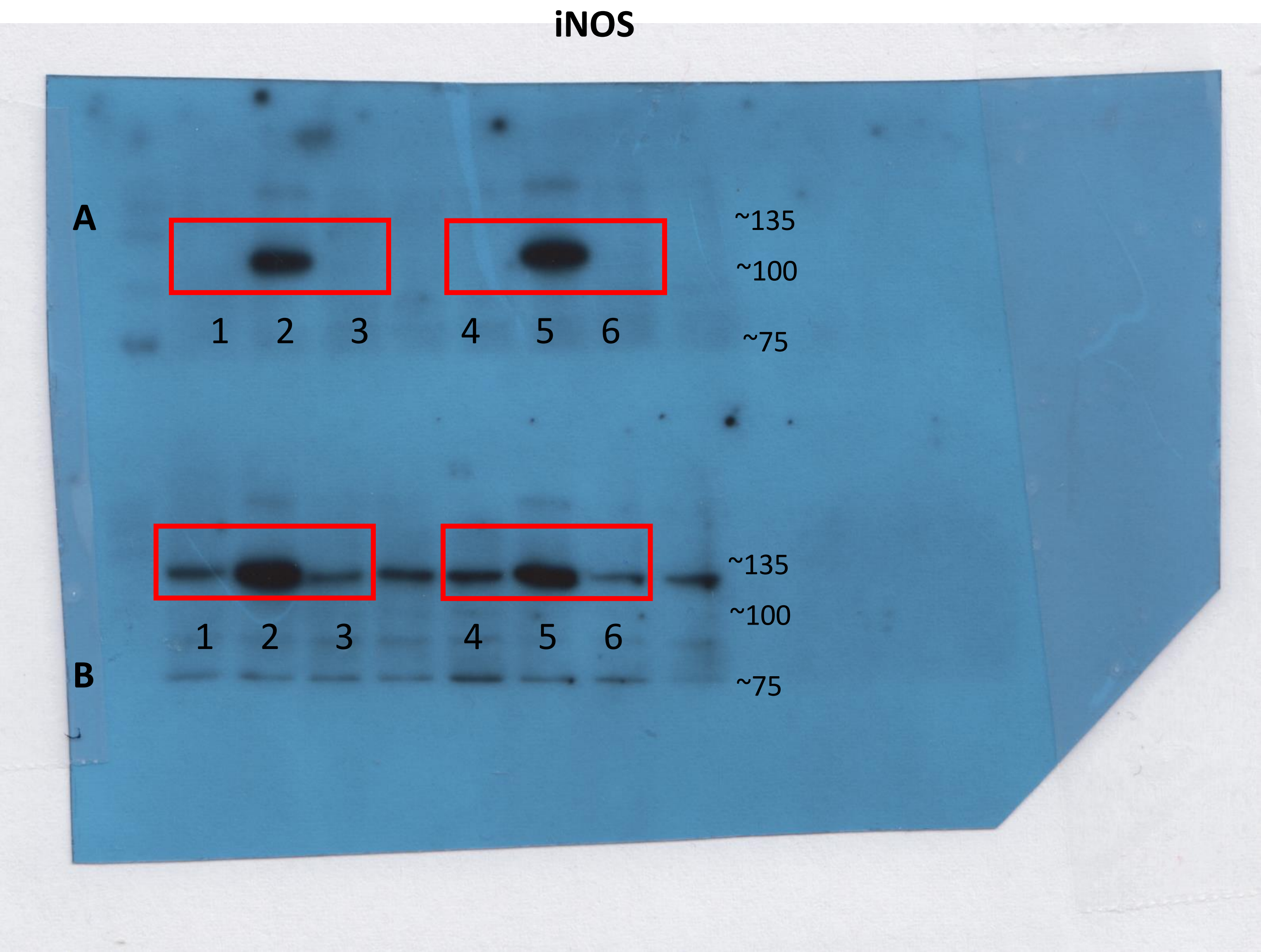

1. NT_{37}
2. LPS_{37}
3. TAK_{37}
4. NT_{39}
5. LPS_{39}
6. TAK_{37}

A) J774.1A cells
B) RAW264.7 cells

Full unedited blot for figure 6

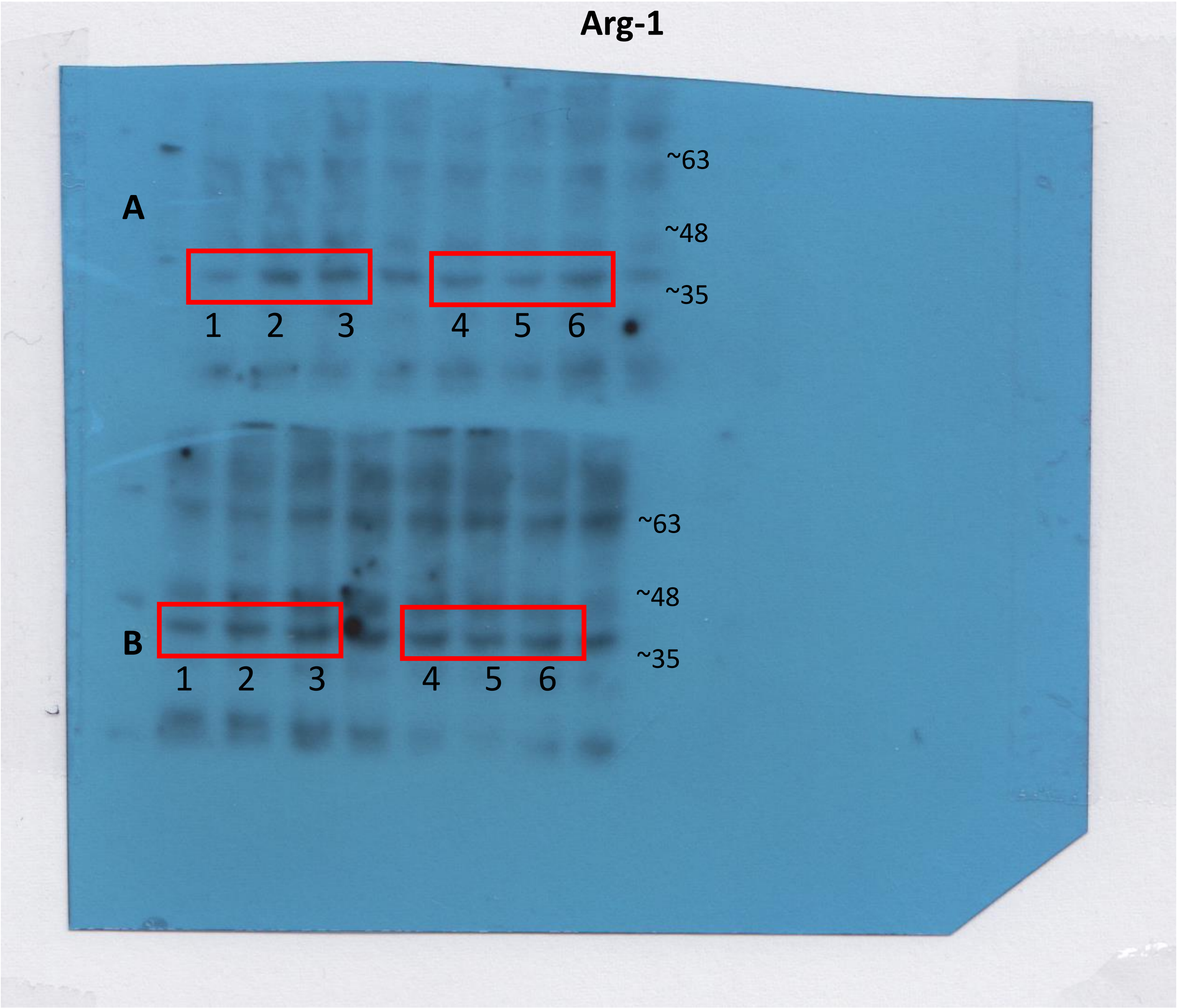

1. NT_{37}
2. LPS_{37}
3. TAK_{37}
4. NT_{39}
5. LPS_{39}
6. TAK_{37}

A) J774.1A cells
B) RAW264.7 cells
